# Single-cell Iso-Sequencing enables rapid genome annotation for scRNAseq analysis

**DOI:** 10.1093/genetics/iyac017

**Published:** 2022-02-10

**Authors:** Hope M Healey, Susan Bassham, William A Cresko

**Affiliations:** 1 Institute of Ecology and Evolution, University of Oregon, Eugene, OR 97403, USA; 2 Presidential Initiative in Data Science, University of Oregon, Eugene, OR 97403, USA

**Keywords:** PacBio, emerging model organisms, 10X Genomics

## Abstract

Single-cell RNA sequencing is a powerful technique that continues to expand across various biological applications. However, incomplete 3′-UTR annotations can impede single-cell analysis resulting in genes that are partially or completely uncounted. Performing single-cell RNA sequencing with incomplete 3′-UTR annotations can hinder the identification of cell identities and gene expression patterns and lead to erroneous biological inferences. We demonstrate that performing single-cell isoform sequencing in tandem with single-cell RNA sequencing can rapidly improve 3′-UTR annotations. Using threespine stickleback fish (*Gasterosteus aculeatus*), we show that gene models resulting from a minimal embryonic single-cell isoform sequencing dataset retained 26.1% greater single-cell RNA sequencing reads than gene models from Ensembl alone. Furthermore, pooling our single-cell sequencing isoforms with a previously published adult bulk Iso-Seq dataset from stickleback, and merging the annotation with the Ensembl gene models, resulted in a marginal improvement (+0.8%) over the single-cell isoform sequencing only dataset. In addition, isoforms identified by single-cell isoform sequencing included thousands of new splicing variants. The improved gene models obtained using single-cell isoform sequencing led to successful identification of cell types and increased the reads identified of many genes in our single-cell RNA sequencing stickleback dataset. Our work illuminates single-cell isoform sequencing as a cost-effective and efficient mechanism to rapidly annotate genomes for single-cell RNA sequencing.

## Introduction

Single-cell RNA sequencing (scRNAseq) is a revolutionary technique in biology that provides expression information from tissues and embryos (Shapiro *et al.* 2013). By barcoding RNA from individual cells directly from dissociated samples, scRNAseq allows for post hoc analysis of cell types and can be used to ascertain novel cell populations, explore developmental trajectories, and define gene regulatory networks (Luecken and Theis 2019).

To maximize the utility of scRNAseq datasets, however, 3′-UTRs must be annotated for several reasons. ScRNAseq captures transcripts through poly(A) tails leading to a 3′ bias in coverage (Hwang *et al.* 2018). Partially annotated genes may be represented in a dataset with lower read counts, leading to erroneous conclusions regarding their magnitude of expression across cell types. In addition, downstream scRNAseq analysis clusters cell types through the determination of a covariance structure across highly variable genes (Stuart *et al.* 2019). The systematic absence of such genes can lead to inferential errors in multivariate analyses and obscure biological reality.

The annotations of even intensively studied models, such as mice and zebrafish, continue to be improved (Gupta *et al.* 2018; Lawson *et al.* 2020). Most other organismal genomes are even less well annotated. Facilitating a broader utility of scRNAseq requires more efficient methods for 3′-UTR annotation. Recently, full length, single-molecule isoform sequencing (Iso-Seq) has been used to improve genome annotations (Kuo *et al.* 2017, 2020; Beiki *et al.* 2019; Ali *et al.* 2021). PacBio’s Iso-seq has been further adapted to use the 10x Genomics platform for scRNA barcoding single-cell isoform sequencing (ScISOr-Seq) to track cell type specific isoform expression (Gupta *et al.* 2018; Zheng *et al.* 2020).

Here, we show that ScISOr-Seq in the context of a scRNAseq experiment allows rapid 3′-UTR annotation in threespine stickleback fish (*Gasterosteus aculeatus*). This fish has long been a focus of study in behavior, ecology, and evolution (Bell and Foster 1994; Colosimo *et al.* 2004; Cresko *et al.* 2004, 2007; Shapiro *et al.* 2004; Hohenlohe *et al.* 2010; Reid *et al.* 2021), and is now a nascent system for biomedical research (Miller *et al.* 2007; Gardell *et al.* 2017; Small *et al.* 2017; Beck *et al.* 2020, 2021; Fuess *et al.* 2021). Although stickleback has a well-assembled genome, its 3′-UTR annotations are incomplete which limits scRNAseq’s utility. We demonstrate that a single PacBio SMRT cell of ScISOr-Seq data is sufficient to significantly improve the stickleback annotations to an extent on par with zebrafish for the purpose of scRNAseq analysis at this stage. Our findings demonstrate that ScISOr-Seq will be a useful tool to efficiently improve genome annotations for scRNAseq in many organisms.

## Materials and methods

### Tissue dissociation to generate a pool of single cells

We crossed a laboratory line of stickleback originally isolated from Cushman Slough (Oregon) and raised embryos to 70 hours post fertilization (hpf) at 20°C using standard procedures from the Cresko Laboratory Stickleback Facility (Cresko *et al.* 2004). 70 hpf stickleback embryos are developmentally at an equivalent stage to 24 hpf zebrafish (Cresko *et al.* 2003). We euthanized 36 embryos in MS-222 following IACUC approved procedures then dechorionated and deyolked them at room temperature. We limited our embryo dissection to 20 min based on Farrell *et al.* (2018). Following protocols from Bresciani *et al.* (2018), we dissociated the cells for 6 min in 0.25% trypsin in PBS at 30°C, pipetting up and down every 30 s, then stopped the dissociation using 10% FBS DMEM and spun down cells at 400xG for 3 min at 4°C. We resuspended cells in 1 ml of 0.1% BSA PBS, centrifuged at 400xG for 3 min at 4°C, and resuspended in 100 µl 0.04% BSA in PBS. At room temperature, we filtered cells through a 40 µM cell strainer (Thomas scientific 1181X52) then washed the original tube twice with 100 µl of 0.04% BSA in PBS and poured over the same cell strainer.

### Library preparation

The scRNAseq and ScISOr-Seq libraries were prepared and sequenced by the University of Oregon Genomics and Cell Characterization core facility (https://gc3f.uoregon.edu). The dissociated cells were diluted to 800 cells/µl in 0.04% BSA in PBS to target 10,000 cells with the 10X Genomics Single Cell 3′ Genome Expression (GEX) mRNA-Seq prep with v3.1 NextGem chemistry. The single 10X preparation was split to create the scRNAseq and ScISOr-Seq libraries. The scRNAseq sample was sequenced on one-seventh of a single S4 lane on a NovaSeq 6000 platform (Illumina). For the ScISOr-Seq library, 400 ng of the amplified 10X cDNA was used as input for the SMRTbell Express Template Prep Kit 2.0 (P/N 100-938-900) without reamplification. Sample specific barcode (ATATAGCGCGCGTGTG) was added using the Barcoded Adapter Kit 8B—OVERHANG (P/N 101-628-500). The ScISOr-Seq library was sequenced on a single SMRT Cell 8M on the PacBio Sequel II platform using the v4 primer, v2.1 polymerase, 1 h binding, 30-h movie, and 2-h pre-extension time at a loading concentration of 100 pM.

### PacBio data processing

The University of Oregon Genomics and Cell Characterization core facility (https://gc3f.uoregon.edu) generated “circular consensus” reads for our ScISOr-Seq dataset (ccs -j 39 –min-passes 3 –min-snr 2.5 –min-length 10 –max-length 50000 –min-rq 0.99; v6.6.0) and used lima (-j 39 –isoseq; v2.2.0) to remove a sample specific barcode (ATATAGCGCGCGTGTG) as a part of the PacBio SMRT Analysis software (Supplementary File 6). After barcode clipping, we used a custom ScISOr-Seq processing script (scISOr_Seq_processing.py) that removed the sample primers (3p: CTACACGACGCTCTTCCGATCT; 5p: CCCATGTACTCTGCGTTGATACCACTGCTT), removed and saved cell and UMI barcodes, removed poly(A) tails, then filtered out duplicated reads. This script outputs reads that have the expected presence and orientation of primers and poly(A) tails for downstream analysis. The script additionally outputs reads without the expected primers in a separate file; however, these other reads were not used for further analysis.

We aligned reads to the stickleback genome (BROAD S1, 104.1 database version) using minimap2 (v2.7; parameters: -ax splice, -uf, -06,24, -B4; (Li 2018). We clustered for unique transcripts with collapse_isoforms_by_sam.py script (c = 0.99, I = 0.95; (Tseng 2021)) from cDNA Cupcake (v27.0.0), classified transcripts with the sqanti3_qc.py script from SQANTI3 (v4.2), and refined transcripts with sqanti3_RulesFilter.py script (Tardaguila *et al.* 2018). We used the sqanti_classification.filtered_lite.gtf that resulted from the refining step (Supplementary File 2) to run Cell Ranger from 10X Genomics (v3.2.0) as described below.

We downloaded the raw data from Naftaly *et al.* (2021) from NCBI. This adult Iso-Seq dataset contains gonad, pronephros, brain, and liver reads from both sexes, a total of 16 SMRT cells. We processed reads from this dataset with the following steps: we generated circular consensus reads with ccs (–min-rq=.9; v6.0.0), clipped barcodes listed in Naftaly *et al.* (2021) with lima (–isoseq –dump-clips; v2.2.0), removed polyA tails with isoseq3 refine (–require-polyA; v3.4.0-0), and clustered reads with isoseq3 cluster (v3.4.0-0) from the PacBio SMRT Analysis software (Supplementary File 6). For the analysis with solely these data, we aligned reads to the stickleback genome (BROAD S1, 104.1 database version), collapsed transcripts with cDNA Cupcake (v28.0.0), and filtered and classified with SQANTI3 (v4.2) as was done with the ScISOr-Seq data. Again, we used the sqanti_classification.filtered_lite.gtf from SQANTI3 (Supplementary File 3) to run Cell Ranger as described below. Due to reduced novel and increased annotated genes relative to Naftaly *et al.* (2021), we additionally tried using minimap2-2.15 with -ax splice -uf -C5 -secondary=no to match their parameters; however, we observed negligible changes. We propose that differences in our analyses arise from different earlier processing steps, different versions of stickleback gene models, and an updated version of SQANTI.

We pooled both sets of Iso-Seq reads and merged it with existing Ensembl annotations to create an improved version of the Ensembl annotations. Previously, we created a modified version of the Ensembl annotations where we extended the 3′-UTRs (Supplementary Files 7 and 8) for several marker genes (*tbx16, sox10, sox32*, and *eya1*) and fgf/fgfr genes (*fgfr1a, fgfr1b, fgfrl1a, FGFRL1, fgfr2, fgfr3, fgfr4, fgf3, fgf4, fgf16, fgf17, fgf6, fgf6a, fgf8a*, and *fgf8b)*. Because we lacked Iso-Seq reads for *fgf4*, we used this modified gtf for the merging (Supplementary File 8). We completed the processing of the ScISOr-Seq and bulk Iso-Seq data separately as described above then combined the files prior to alignment. We aligned the combined files with minimap2, collapsed transcripts with cDNA cupcake, and refined and classified isoforms with SQANTI3 as explained above.

We merged the sqanti_classification.filtered_lite.gtf resulting from SQANTI3 with the Ensembl annotation using TAMA (Kuo *et al.* 2020). To prepare the gtf files for TAMA Merge, we converted them to bed files using bedparse gtf2bed (–extraFields gene_id). Then, modified the output with awk to rearrange the columns of the bed file such that the gene id was separated from the transcript id by a semicolon (awk -v OFS=‘\t’ ‘{print $1,$2,$3, $13 “;” $4, $5, $6,$7,$8,$9,$10,$11,$12}’). We merged with TAMA’s script tama_merge.py (-s ensembl -cds ensembl -d merge_dup). We applied the ensembl gene names to genes with corresponding IDs using a custom script (tama_associating_ensembl_ids_with_genes.py). For our final gtf file, we converted the output bed file back to a gtf with TAMA’s tama_convert_bed_gtf_ensembl_no_cds.py (Supplementary File 4). We also generated a gtf file specifically for scRNAseq analysis where mitochondrial genes started with “MT” by transforming the bed file with a custom script (tama_associating_ensembl_ids_with_genes_for_scRNAseq.py) and then converting the output back to a gtf in the same fashion as above (Supplementary File 5).

### Annotation analysis

We assessed the Iso-Seq improvements to Ensembl gene models in 2 ways: (1) differences in starting and ending positions and (2) differences in reads captured. We compared the 5′ start and 3′ end of Ensembl and Iso-Seq generated gene models with a custom script written by H.M.H. (tama_observations_bed.py). Using the same custom script, we also compared the number of reads present in the first and last exons between Ensembl and Iso-Seq gene models.

### scRNAseq analysis

We quantified gene counts using 10X Genomics Cell Ranger v3.0.2 (Supplementary File 1). We generated a reference using mkgtf and mkref, retaining protein coding and nonprotein coding genes, and then aligned and counted reads using the stickleback genome (BROAD S1, 104.1). We analyzed the counts using the Seurat package [v3.2.3; (Stuart *et al.* 2019)] on R (v4.0.2). We retained all cells for the analysis. We normalized the counts using SCTransform and regressed out the mitochondrial genes using the glmGamPoi method (Hafemeister and Satija 2019). Based on the inflection point in the elbow plot of the PCA results, we chose 38 dimensions for generating the UMAP and identifying clusters. We identified cluster identities using 3 marker gene approaches.

First, we searched for marker genes identified in Farnsworth *et al.* (2020) for specific cell types found in a similar developmental stage from zebrafish (24 hpf in zebrafish). Next, we identified markers with Seurat’s FindAllMarkers searching for genes that were positively upregulated in each cluster compared to the other identities, in a minimum of 25% of cells, and a log fold change threshold of 0.25. Finally, we identified markers with Seurat’s FindMarkers searching for genes that were positively upregulated in each cluster vs all the other cells. For the second and third approaches, we searched for the homologous gene in zebrafish and used expression data from zfin to predict which cell types expressed the gene. To quantify *frizzled* gene counts, we used FetchData to isolate raw counts for each gene and the number of cells expressing the gene. To determine the number of cell clusters each *frizzled* gene was expressed in, we saved a dotplot, subsetted for clusters where the percentage of cells expressing the gene was greater than 10%, and then counted the number of clusters left for each gene.

## Results and discussion

### ScISOr-Seq captured novel isoforms and improved 3′-UTRs

**Fig. 1.**
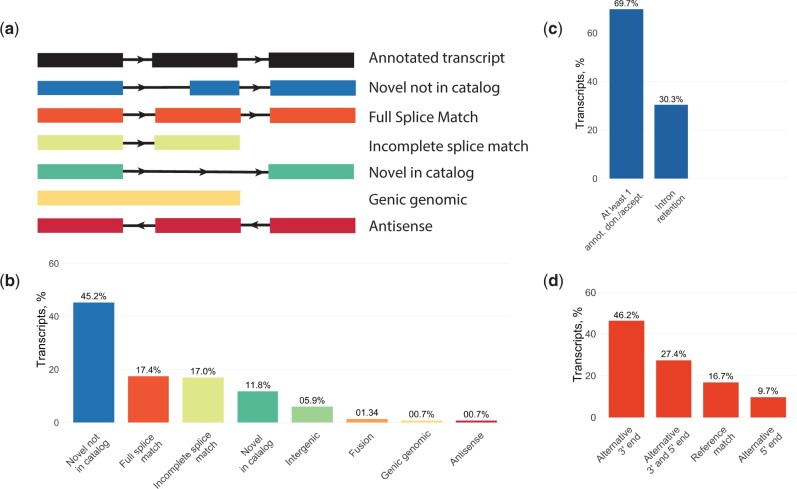
SQANTI3 classification of ScISOr-Seq data revealed that the majority of isoforms were previously unannotated in the stickleback genome. a) SQANTI3 categorizes isoforms based on their match to the reference, the major categories are shown here [not shown: Fusions (isoform contains 2 original separate gene models) and Intergenic (isoform present in intergenic regions)]. b) The distribution of ScISOr-Seq isoforms in the major SQANTI3 classes illustrating that the most common class of isoforms are novel not in catalog. c) Within the novel not in catalog category, isoforms are largely coming from cases where there is at least 1 new splicing donor or acceptor with some coming from cases of intron retention (for example, nkx2.3 isoforms from Fig. 3). d) Nearly half of isoforms that are full splice matches have at least an alternative 3′ site while few are complete reference matches.

A ScISOr-Seq library was produced using dissociated cells from 70 hpf stickleback embryos. The ScISOr-Seq reads were classified with SQANTI3 (Tardaguila *et al.* 2018) using Ensembl gene models to describe isoforms based on how well they match Ensembl splice variants (Fig. 1a). The most common structural category (45.15% of isoforms) was “novel not in catalog” containing at least 1 new splicing site relative to the existing annotation (Fig. 1, a and b). 17.42% of collapsed isoforms were “full splice matches” (FSM) that contained splicing sites and exons present in the Ensembl annotation but allowed differences in 5′ or 3′ ends. Notably, only 3% of the unique isoforms matched the existing gene annotations, while 97% improved existing or added gene models. Confirming that 3′-UTRs were poorly annotated, 46.2% FSM isoforms had alternative 3′ ends and 27.4% had alternative 3′ and 5′ ends relative to Ensembl gene models (Fig. 1c). Therefore, the existing stickleback annotation—in addition to having incomplete 3′-UTRs—is also missing many splice variants. Our work indicates that additional ScISOr-Seq or bulk Iso-Seq is necessary to capture these variants as well as prune erroneous transcript models from the Ensembl annotation.

### ScISOr-Seq improved the number of reads retained for scRNAseq

**Fig. 2.**
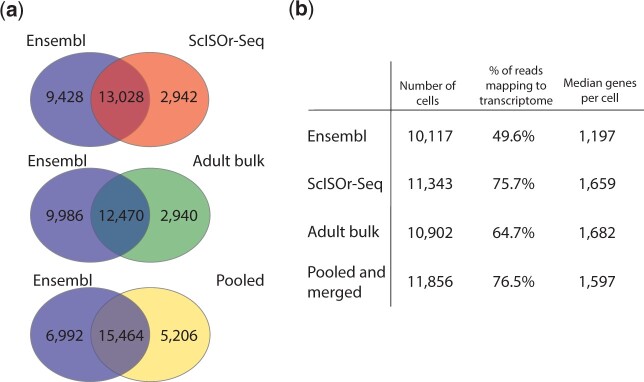
ScISOr-Seq (70 hpf) and bulk Iso-Seq (gonad, brain, liver, and pronephros) captured a limited number of annotated transcripts, but both together resulted in an increase in reads mapping to the transcriptome. Ultimately, pooling these data and merging with the Ensembl annotation resulted in the largest gains in reads mapped. a) The number of genes in common and unique to each Iso-Seq read category is compared to the Ensembl genome. b) Results from Cell Ranger runs using the different genome annotation files illustrating the overall improvements by using Iso-Seq data particularly the ScISOr-Seq gene models.

We initially tested how well the existing stickleback gene models from Ensembl (BROAD S1, 104.1 database version) would capture scRNAseq reads. Strikingly, less than 50% of reads were retained for downstream scRNAseq analysis (Fig. 2b; Supplementary File 1). Using such an incomplete annotation for scRNAseq would likely result in erroneous interpretation of gene expression patterns for genes lacking 3′-UTR annotations.

**Fig. 3.**
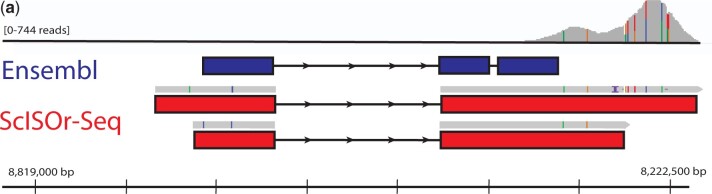
ScISOr-Seq improved gene models by extending the 3′-UTRs illustrated here by the 2 models for *nkx2.3*. ScRNAseq reads are shown at the top in gray, the scale of reads is to the left of them. The existing Ensembl model (ENSGACG00000007568; ENSGACT00000010057.1) is shown in dark blue. Notably, this model does not overlap with the majority of the scRNAseq reads. The ScISOr-Seq reads (in gray) are shown above the respective gene models (in red) that they generated. Colored lines on the ScISOr-Seq and scRNAseq reads indicate a different base pair in the read than the stickleback reference genome (BROAD S1, 104.1 database version). The scale bar at the bottom indicates the position of the *nkx2.3* gene models on group VI. The ScISOr-Seq gene models captures all the scRNAseq reads.

Next, we used ScISOr-sequencing data to generate new gene models and tested how well these new models would capture 10X based illumina scRNAseq reads (Supplementary File 2). Using the ScISOr-Seq dataset, 13,028 of 22,456 previously annotated genes and 2,942 novel genes were identified (Fig. 2a). The ScISOr-Seq annotations lead to a notable 26.1% increase in reads retained from scRNAseq compared to the Ensembl gene models alone (Fig. 2b; Supplementary File 1). The alignment of scRNAseq reads with existing Ensembl and new ScISOr-Seq gene models illustrates that ScISOr-Seq models retain greater numbers of reads due to improved annotation of 3′-UTRs (Fig. 3; Supplementary Fig. 1). This result compares favorably with current research in the much better studied zebrafish model. Farnsworth *et al.* (2020) completed their zebrafish atlas with 80% of reads retained in 5 dpf (days post fertilization) fish. After Lawson *et al.* (2020) improved the zebrafish transcriptome using RNAseq data, they noted a 4% increase in reads retained that led to an increase of 2,257 cells and 8 clusters using the same 5 dpf atlas (Farnsworth *et al.* 2020). The similarity in number of retained reads in our dataset with those published in zebrafish improves the validity of stickleback scRNAseq investigations.

Recently, Naftaly *et al.* (2021) published a large bulk analysis of adult stickleback Iso-Seq data from 16 PacBio SMRT cells and 4 tissue types (gonad, brain, pronephros, and liver). We reanalyzed this dataset to test whether we would see comparable improvements in our scRNAseq analysis (Supplementary File 3). Although we identified similar overall annotated and novel genes, the bulk Iso-Seq annotation from adult stickleback captured 11% fewer reads than our embryonic ScISOr-Seq annotation (Fig. 2, a and b; Supplementary File 1). These results are likely due to differential expression of transcripts between the adult and embryonic samples and highlight that even for species with previous Iso-Seq libraries, additional sequencing may be needed for scRNAseq analysis in other developmental stages or tissue types.

**Fig. 4.**
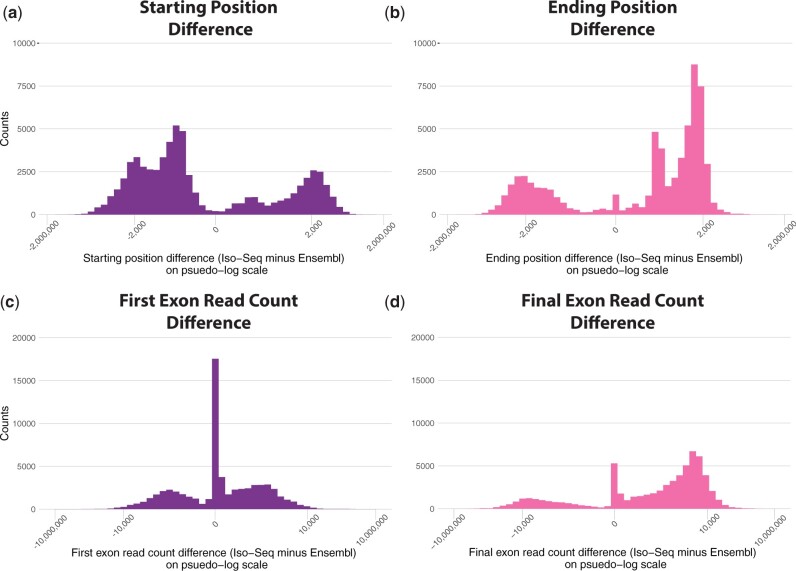
Annotation improvements using Iso-Seq (bulk Iso-Seq or ScISOr-Seq) data extended genes both 5′ and 3′, with 3′ expansions leading to greater numbers of reads counted. Comparing the median difference in starting or ending position between the Ensembl annotations and pooled Iso-seq annotations shows that bulk Iso-Seq and ScISOr-Seq reads overall lead to earlier starting positions (median = −26, a) and extended ending positions (median = 94, b). These Iso-Seq differences increased the number of reads counted in the final exon of the gene (median = 341, d); however, the alterations led to negligible changes of reads counted in the first exon (median = 0, c), which highlights the 3′ bias of scRNAseq reads. The *x*-axis is plotted on a pseudo log scale to account for the negative values.

Because both our ScISOr-Seq and the bulk Iso-Seq gtf files improved the number of scRNAseq reads retained, we pooled them to create a comprehensive annotation and also merged this pooled dataset with the Ensembl annotations. We retained all transcripts and united them under a single gene model for scRNAseq analysis (Supplementary File 4). The Iso-Seq generated gene models extended 3′ ends of transcripts and increased reads counted in the final exon (Fig. 4). This new annotation contained 6,992 genes unique to Ensembl, 15,464 genes containing transcripts from Ensembl and Iso-Seq, and 5,206 genes unique to the pooled Iso-Seq dataset (Fig. 2a). Since reads are filtered during analysis, the 6,992 genes unique to Ensembl might still be expressed in 70 hpf embryos or the adult tissues. For instance, 2,152 Ensembl annotated genes were removed from the pooled Iso-Seq dataset by SQANTI3’s intra-priming and template switching filter. Although pooling and merging added 16 SMRT cells of Iso-Seq data as well as the full suite of Ensembl annotations, this effort only improved the proportion of reads retained by +0.8% beyond the annotation created by just our single ScISOr-Seq sample (Fig. 2b; Supplementary File 1). Therefore, the gene models originating from a matching ScISOr-Seq library alone could be used for scRNAseq analysis if a system lacks a prior annotation.

### ScISOr-Seq enhances biological interpretation of cell clusters

**Fig. 5.**
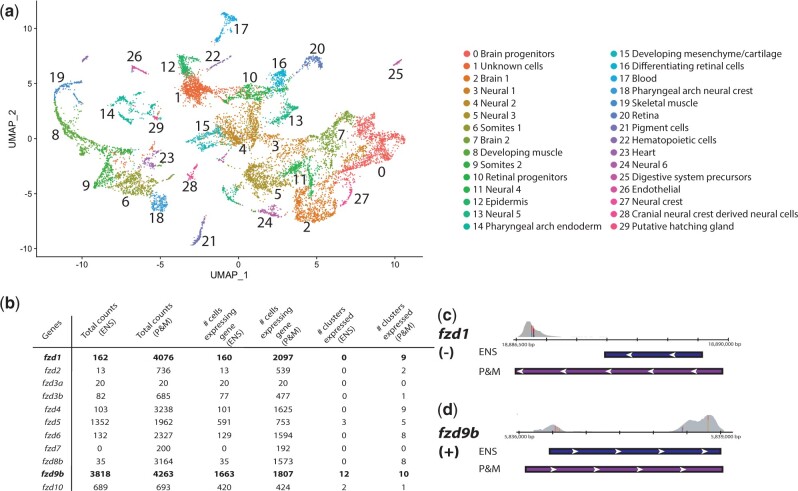
Using the updated gene models from our merged and pooled dataset, we identified expected cell types in 70 hpf stickleback scRNAseq data and illustrated how our annotation improvements are relevant for analysis. a) The clustering and cell identities of the scRNAseq data are illustrated on the UMAP. b) Frizzled gene family expression compared between the Ensembl annotation scRNAseq analysis and the pooled and merged analysis using total raw counts, number of cells expressing each gene, and the number of clusters where over 10% of cells are expressing each gene. c) *fzd1* gene model is greatly extended in the pooled and merged annotation (purple) compared to the Ensembl model (blue) which allows for much greater counting of reads (gray). d) *fzd9b* had minor changes in the pooled and merged model (purple), a longer 5′-UTR, in comparison with the Ensembl annotation (blue) which led to minimal increases in reads (gray) counted. The scRNAseq read pileups in c and d have colored lines on base pairs that are different from the stickleback reference genome (BROAD S1, 104.1 database version).

Using the pooled (ScISOr-Seq and bulk Iso-Seq) annotation that was merged with Ensembl annotation, we proceeded with scRNAseq analysis (Supplementary File 5) to test whether biologically meaningful cell clusters would be created. We clustered cells by their transcriptional profiles into cell identities and analyzed the results with Seurat (Stuart *et al.* 2019). We identified 30 clusters of cells with 38 principal components (Fig. 5a). We putatively annotated cell types *via* distinguishing sets of marker genes based on zebrafish literature (Howe *et al.* 2013; Wagner *et al.* 2018; Farnsworth *et al.* 2020).

Although zebrafish and stickleback diverged ∼229.9 MYA (Howe *et al.* 2021), we identified similar cell types as observed in existing zebrafish atlases for similar developmental stages as we analyzed in stickleback (Wagner *et al.* 2018; Farnsworth *et al.* 2020). In addition, we compared the expression of *sox9a* and *sox9b* in our atlas to published descriptions of stickleback in situ expression at the same stage (Cresko *et al.* 2003). Supporting our cluster annotations, we observed expression of both SOX genes in roughly the same cell types as defined by in situ hybridization (Supplementary Fig. 2, a and b; Cresko *et al.* 2003). Despite our success using a single SMRT cell for ScISOr-Seq for isoform annotation, we would need to sequence additional SMRT cells to have enough data to correlate cell types and specific isoforms.

Since the pooled and merged dataset has higher numbers of cells, genes per cell, and total genes detected than the Ensembl scRNAseq dataset (Fig. 2b), assessing the degree to which pooled and merged gene models improved the scRNAseq analysis is complex. For instance, a higher overall expression of a gene in a cluster might be the result of different clustering patterns, more cells retained, and/or more reads counted. We compared the raw counts of specific genes, number of cells expressing these genes, and number of clusters where at least 10% of cells expressed them (Fig. 5b). Since Cell Ranger provided a subset of these values on a global scale (Fig. 2b), we chose to examine changes in gene counts, cell number, and number of clusters with a case study: the *frizzled* genes, a family of Wnt-pathway signaling molecules expressed in a wide range of tissues (Huang and Klein 2004; Wang *et al.* 2016). For 10 of 11 *frizzled* genes, we observed increases in raw counts and number of cells expressing them in the pooled and merged annotation (Fig. 5b). Some genes, however, exhibited dramatic increases (*fzd1, fzd2, fzd3b, fzd4, fzd6, fzd7*, and *fzd8b*) while others exhibited minor increases (*fzd5, fzd9b*, and *fzd10*). Comparing the gene models of *fzd1* and *fzd9b*, we determined which factors influenced expression detection. Importantly, the 5′- and 3′-UTRs expanded for *fzd1* in the merged and pooled annotation, however only the 5′ end of *fzd9b* was extended (Fig. 5, c and d). Based on the read alignments (Fig. 5, c and d), the increase in counted reads for *fzd1* was due solely to 3′-UTR changes, while a slight increase in *fzd9b* reads was due to 5′ end extension. In addition to increased read counts, 7 of 11 genes were expressed in more cell clusters in the pooled and merged dataset. Two genes, *fzd9b* and *fzd10*, were found in 1 less cluster. We hypothesized that 2 clusters (containing *fzd9b* and *fzd10*) were combined in the merged and pooled dataset relative to Ensembl. Overall, this gene family comparison illustrated the problem of using an annotation with incomplete 3′-UTRs. If the Ensembl dataset’s *fzd* counts were accepted as their true qualitative and quantitative expression, flawed conclusions would have been made regarding the contributions of each gene in the family.

## Summary

Incomplete 3′-UTR annotations can hinder single-cell transcriptional profiling studies. In addition to reducing the overall number of genes included in an analysis, systematic differences in preliminary UTR annotations could lead to significant inferential errors. We illustrated in stickleback fish that a minimal ScISOr-Seq dataset, generated concurrently with scRNAseq data, was capable of dramatic improvements in retained read counts (+26.1%). In addition, we showed that pooling additional Iso-Seq reads from adult bulk samples and merging with existing Ensembl annotations improved scRNAseq reads retained negligibly beyond models solely from embryonic ScISOr-Seq (+0.8%). Using the improved annotation for scRNAseq permitted identification of cell types and increased the observed expression of numerous genes. Overall, our work illustrates that ScISOr-Seq is a rapid and cost-effective method to annotate genomes of various organisms for scRNAseq and can improve efficacy of biological inferences.

## Data availability

All raw sequencing data associated with this study will be available upon peer-reviewed publication via the NCBI Sequencing Read Archive, under BioProject PRJNA797645. Annotation and summary files in the Supplementary Information will also be available in the Dryad repository (https://doi.org/10.5061/dryad.0k6djhb1x). Supplementary files are available on figshare: https://doi.org/10.25386/genetics.16811614. All scripts can be found in the github repository (https://github.com/CreskoLab/scISOseq_processing).
